# Immune Reactivity in Hippocampal Subregions of Cognitive SuperAgers with Primary Age‐related Tauopathy

**DOI:** 10.1002/alz70861_108955

**Published:** 2025-12-23

**Authors:** Caroline M Nelson, Ivan Ayala, Antonia Zouridakis, Grace Minogue, Allegra Kawles, Rachel M Keszycki, Alyssa Macomber, Molly A Mather, Pouya Jamshidi, Sandra Weintraub, Rogan A Grant, Rudolph J Castellani, Marsel Mesulam, Changiz Geula, Tamar Gefen

**Affiliations:** ^1^ Mesulam Center for Cognitive Neurology & Alzheimer's Disease, Chicago, IL USA; ^2^ Division of Pulmonary and Critical Care Medicine, Feinberg School of Medicine, Northwestern University, Chicago, IL USA

## Abstract

**Background:**

Cognitive SuperAgers are individuals age 80+ with episodic memory equal to individuals 20‐30 years their junior. Prior investigations of this cohort revealed unique neuropathologic features compared to same‐aged cognitively‐average peers including less microglia activation in white matter and lower overall neurofibrillary degeneration. Primary age‐related tauopathy (PART)— which is confined to medial temporal lobe structures and absent of amyloid‐β plaques—is observed at relatively high frequency in SuperAgers. Here we examined signatures of glial immunoreactivity in participants to identify putative neuroinflammatory factors contributing to the SuperAging phenotype.

**Method:**

Eight cases with postmortem PART were identified from Northwestern ADRC brain bank (*N* =4 SuperAgers; N=4 age‐matched cognitively‐average controls). Paraffin‐embedded hippocampal sections were immunohistochemically stained with AT‐8 (pTau accumulation), GFAP (astrocytic reactivity), HLA‐DR (proinflammatory microglia), and Olig2 (oligodendrocytes). Total staining reactivity (% area covered) was quantified from 20X magnification digital images using QuPath software (v.0.5.1) across the CA1, CA2, CA3, and dentate gyrus (DG) of the hippocampal complex. One‐way ANOVA examined differences between stains, and two‐way ANOVAs analyzed regional and group differences.

**Result:**

No differences were observed in the percent area immunoreactivity of pTau or proinflammatory microglia between SuperAgers and controls nor across hippocampal subregions. Oligodentrocytic reactivity was significantly lower in SuperAgers than controls (*p* <.001). Across all cases, astrocytic immunoreactivity was substantially higher compared to pTau (*p* <0.001), oligodentrocytic reactivity (*p* =.002), and microglia (*p* =.003). Astrocytic immunoreactivity differed across hippocampal subregions (*p* <.001) and was significantly higher in SuperAgers than controls (p <.001). SuperAgers also exhibited higher astrocytic reactivity in DG (*p* =.008) compared to controls. There was a significant interaction effect for group and subregion on observed astrocytic immunoreactivity (*p* =.007). Interestingly, across both groups, pTau tended to be highest in the CA1 and CA2, followed by CA3 and DG; an inverse regional pattern was observed in astrocytic reactivity where significantly lower reactivity was apparent in CA1 and CA2 followed by CA3 and DG.

**Conclusion:**

Preliminary findings suggest that compared to cognitively‐average controls, SuperAgers with PART display lowered oligodentrocytic and heightened astrocytic hippocampal response, suggesting a unique neuroinflammatory pattern. Further ongoing investigations in larger cohorts will address hippocampal subfield distributions of glial reactivity in the presence of amyloid‐β.

